# Cross-Linking With Diamine Monomers to Prepare Graphene Oxide Composite Membranes With Varying D-Spacing for Enhanced Desalination Properties

**DOI:** 10.3389/fchem.2021.779304

**Published:** 2021-11-26

**Authors:** Hong Ju, Jinzhuo Duan, Haitong Lu, Weihui Xu

**Affiliations:** School of Materials Science and Engineering, China University of Petroleum, Qingdao, China

**Keywords:** graphene oxide, diamine monomers, d-spacing, salt rejection, desalination

## Abstract

As a new type of membrane material, graphene oxide (GO) can easily form sub-nanometer interlayer channels, which can effectively screen salt ions. The composite membrane and structure with a high water flux and good ion rejection rate were compared by the cross-linking of GO with three different diamine monomers: ethylenediamine (EDA), urea (UR), and p-phenylenediamine (PPD). X-ray photoelectron spectroscopy (XPS) results showed that unmodified GO mainly comprises π-π interactions and hydrogen bonds, but after crosslinking with diamine, both GO and mixed cellulose (MCE) membranes are chemically bonded to the diamine. The GO-UR/MCE membrane achieved a water flux similar to the original GO membrane, while the water flux of GO-PPD/MCE and GO-EDA/MCE dropped. X-ray diffraction results demonstrated that the covalent bond between GO and diamine can effectively inhibit the extension of d-spacing during the transition between dry and wet states. The separation performance of the GO-UR/MCE membrane was the best. GO-PPD/MCE had the largest contact angle and the worst hydrophilicity, but its water flux was still greater than GO-EDA/MCE. This result indicated that the introduction of different functional groups during the diamine monomer cross-linking of GO caused some changes in the performance structure of the membrane.

## Introduction

The shortage of water resources has become a global problem. To solve the growing freshwater crisis, sewage treatment (Shannon et al., 2008; Ren et al., 2020) and seawater desalination technology (Wu et al., 2007; Logan Elimelech, 2012; Shao et al., 2017; Zhao et al., 2017) have been rapidly developed. Membrane filtration and separation technology was rapidly developed due to its low cost, low energy consumption, simple operation, and lack of secondary pollution. To enable the rapid and widespread application of this technology, the development of membrane materials with specific separation functions has become the primary goal of researchers (Rajaeian et al., 2013; Ali et al., 2016; Hosseini et al., 2017; Sun et al., 2020).

In recent years, graphene, a type of two-dimensional carbon nanomaterial, has attracted wide attention in the fields of physical chemistry and material science due to its high specific surface area and excellent electrical and mechanical properties. Graphene oxide (GO), a derivative of graphene, has broad development prospects in molecular separation and desalination due to its excellent molecular permeability (Surwade et al., 2015; Rollings et al., 2016; Zhao et al., 2016; Hou et al., 2018; Li et al., 2018; Guo et al., 2021; Xu et al., 2021).

Nanosheet layers of GO can be stacked on each other to form a layered structure, including a channel between the nanolayers, allowing for the screening of molecules and ions at the nanometer scale due to the selectivity of the nanostructures (Koenig et al., 2012; Liu et al., 2015; Azelee et al., 2017; Shan et al., 2019; Li Z. et al., 2020; Li N. et al., 2020). A GO sheet contains a large number of oxygen-containing groups: carboxyl groups and hydroxyl groups are located around the edges, while carbonyl and epoxy groups are located at the center (Lerf et al., 1998; Suk et al., 2010), which can be cross-linked with diamine monomers to prepare GO composite films with varying d-spacing. The presence of these hydrophilic oxygen-containing groups gives the graphene oxide good hydrophilic properties, and the unique nanostructures form an sp2 nanocapillary network to achieve ultra-fast water molecule transmembrane transport (Eda and Chhowalla, 2010; Loh et al., 2010; Nair et al., 2012; Yu et al., 2020). In addition, the properties of GO membranes are affected by the diffusion mechanism (Wijmans and Baker, 1995), which depends on the GO interlayer spacing. When the GO membrane is placed in an aqueous solution, the water molecules diffuse into the whole membrane, inserting 2–3 water molecules between the individual GO nanosheets, which results in an increase in the spacing between the GO nanosheets. The expanded layer spacing is a challenge for the application of GO films in ion sieving and desalination (Nicolai and Meunier, 2014; Hegab and Zou, 2015; You et al., 2015; Kang et al., 2018; Kang et al., 2019).

To improve the desalination performance of graphene oxide and create a composite membrane with good performance, researchers have studied many aspects of GO, including covalent cross-linking (Hung et al., 2014; Park et al., 2016), physical limitations (Loh et al., 2010), and the partial reduction of GO (Liu et al., 2011). Abraham et al. (Abraham et al., 2017) prepared graphene oxide in different saturated ion solutions and then used Stycast 1,266 epoxy resin to gel the stacked nanosheets to physically control the layer d-spacing to prepare a precise ion-sieved GO membrane. Experiments have shown that when the d-spacing is between 9.8 Å and 6.4 Å, the permeation rate decreases exponentially with the decrease in d-spacing and the final experiment achieved up to a 97% NaCl rejection. Chen et al. (Chen et al., 2017) proved that the layer spacing of GO films can be precisely controlled according to the diameter of the hydrated ions. By controlling the change in the interlayer distance by adding different kinds of cations, other kinds of sieves with larger hydrated volume ions were effectively realized; the most stable adsorption sites of various cations were confirmed by a molecular dynamics simulation. The method of depositing carbon nanotubes on the GO nanosheet layer resulted in good ion screening and a high water permeability, which allows for a simple deposition rate control to prepare ultra-thin high-performance GO membranes for applications in water treatment (He et al., 2018).

For the liquid phase mass transfer of GO films, Mi (Mattevi et al., 2009) proposed multi-functionalization by regulating the layer spacing of GO films by 1) partially reducing GO films with a layer spacing of less than 0.7 nm (Na^+^ hydration radius of 0.36 nm), to achieve water desalination; 2) inserting a large rigid functional group or a soft polymer chain to achieve a GO film layer spacing of 1–2 nm to perform sewage purification and reuse, as well as drug and fuel separation applications; and 3) inserting more large nanoparticles or nano-fibers to make the GO film layer spacing greater than 2 nm, enabling biomedical applications such as artificial kidneys and hemodialysis.

## Experimental

### Materials

Expanded graphite powder (325 mesh) was used as received. The other chemicals used in our work were ethylenediamine (EDA), urea (UR), p-phenylenediamine (PPD), NaNO_3_ (AR), KMnO_4_ (AR), H_2_SO_4_ (AR), H_2_O_2_(35%), HCl (35%), NaCl (AR), and CaCl_2_ (AR).

### Preparation of the GO Membranes

GO was prepared by the laboratory-modified Hummers method. The specific preparation process was as follows:

a 2-g amount of graphite powder, 1 g NaNO_3_, and 12 g KMnO_4_ were weighed, and then placed in 80 ml of 98% concentrated sulfuric acid in a beaker. This was placed in a water bath and cooled with ice water while constantly stirring. The weighed graphite powder and NaNO_3_ were added, and KMnO_4_ was slowly added while the temperature was kept below 10°C. Then the beaker was placed in a constant temperature water bath at 35°C while being stirred continuously. It was then taken out at room temperature and diluted with 160 ml of deionized water. After 15 min, 500 ml of the deionized water at 75°C and 30 ml of H_2_O_2_ were added to remove the excess oxidant. After washing to neutrality, the precipitate was dispersed in water for 2 h. The supernatant was centrifuged to freeze the dried fraction and obtain dried GO powder.

**FIGURE 1:**
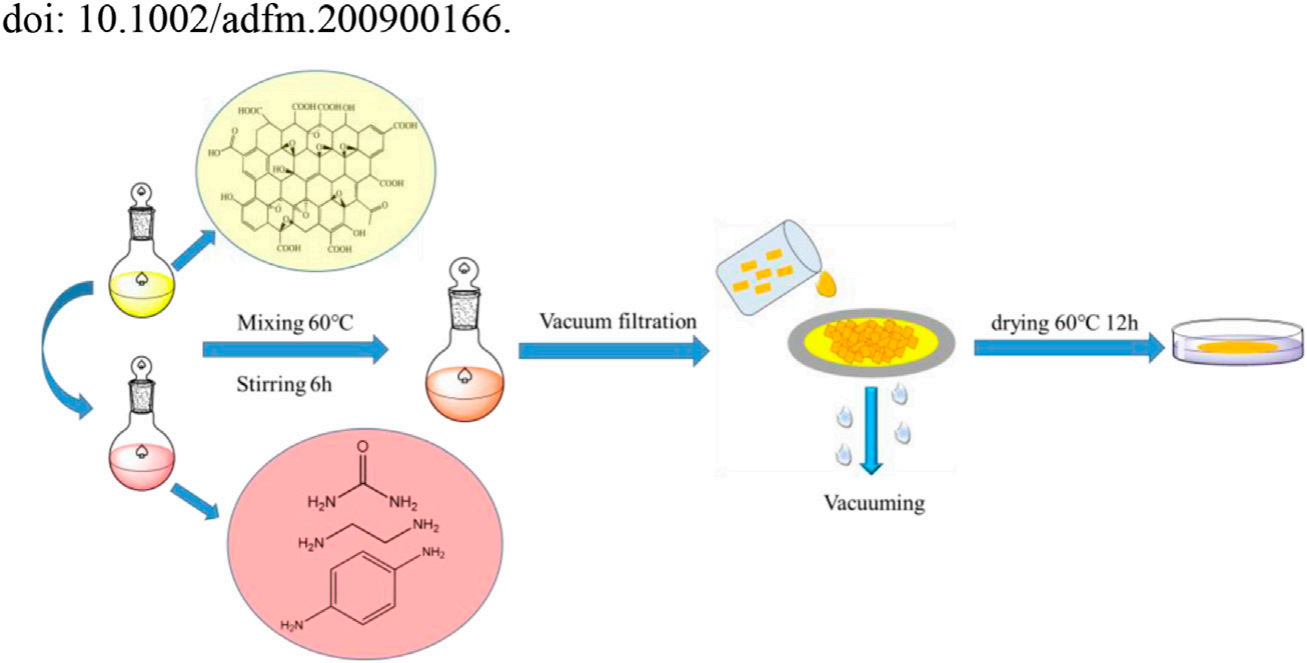
GO composite membranes formation process.

Figure 1 shows the GO composite membrane formation process. The preparation of the urea (UR), ethylenediamine (EDA) and p-phenylenediamine (PPD) crosslinked composite membranes was as follows:

A total of 0.05 mol of a crosslinking agent (UR, EDA, PPD) was added to the 400 mg/L GO suspension, and the mixture was stirred continuously at 60°C for 6 h to sufficiently crosslink the GO and the crosslinking agent.

Each GO, or the crosslinking agent, was then diluted to 10 mg/L, and different volumes of GO or crosslinking agent solutions were added to deionized water to dilute to 200 ml and to obtain diluents containing different quantities of GO or crosslinking agent. (0.01, 0.03, 0.05, 0.10, 0.15 and 0.20 mg). Then, each GO or crosslinking agent was passed through a commercial mixed cellulose (MCE) membrane used as a filter in a vacuum suction device at a fixed pressure of 0.1 MPa to obtain the desired modification of the original GO on the MCE membrane. The area of the membrane was 0.001257 m^2^ (d = 4 cm), and GO and its composite membranes with different deposition mass per unit volume were obtained (7.96, 23.87, 39.79, 79.58, 119.37, and 159.15 mg/m^2^). In order to remove the physical adhesives and redundant cross-linking agents, the composite membrane was immersed in pure water for 12 h.

### Desalination Experimental Device

**FIGURE 2:**
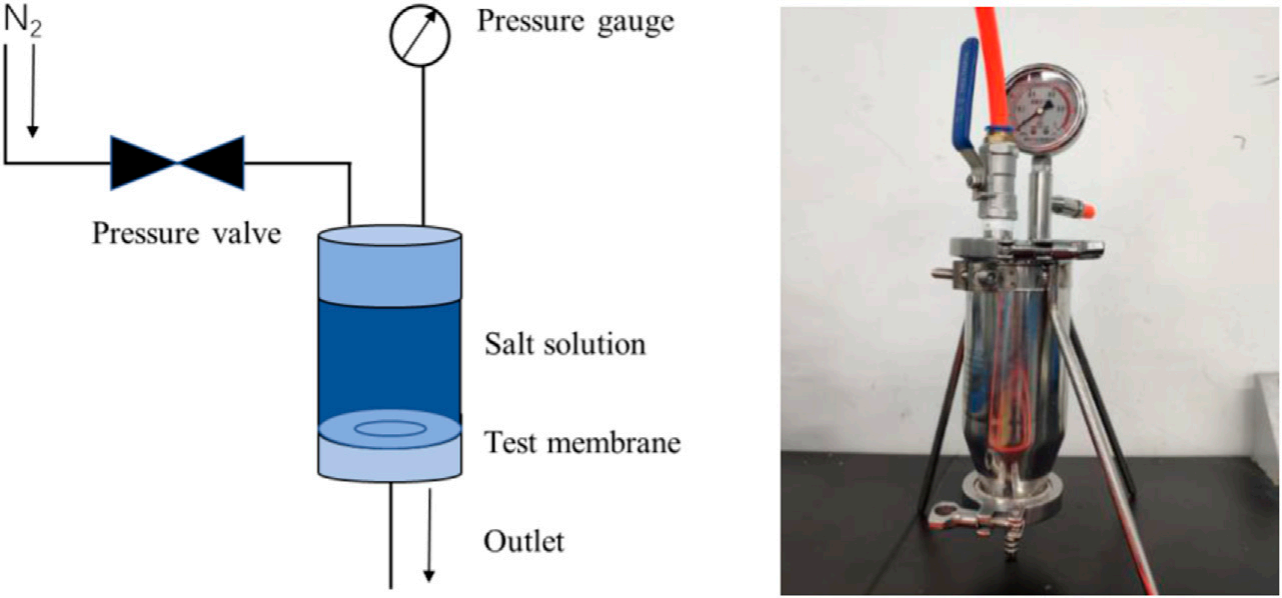
Schematic diagram **(A)** and physical photo **(B)** of positive pressure driven filtration experimental device.

A dead-end filtration system was self-made in the laboratory, and the desalination performance of the composite membrane was tested using 0.05 mol/L NaCl and CaCl_2_ solutions. As shown in Figure 2, a perforated steel plate was first placed on the base of the experimental device, and then a GO reverse osmosis membrane was arranged for testing. The membrane was held in place by an O-ring and the device was sealed. Before closing the valve, 200 ml of salt solution was injected into the container and pressurized with nitrogen through a plastic tube. The pressure used during the experiment was 0.1 MPa. The filtered solution flowed from the lower filter, while the concentrate remained in the container.

The water flow rate was calculated by the following equation:

F=V/Pst, (1)

where F is the water flow rate (L/m^2^·hbar); V is the permeation volume (L); s is the total pressure permeation area (m^2^); t is the penetration test time (h); and P represents the suction pressure (bar, 1bar = 0.1 MPa).

The membrane permeation volume is calculated by the following equation:

Q=Ft, (2)

where Q is the permeation volume (L/m^2^·bar); F represents the water flow rate (L/m^2^·h bar); and t is the permeation time(h).

The desalination rate is calculated by the following formula:

$$\text{R}=\left(1-\frac{\text{C}2}{\text{C}1}\right)100\%,$$ (3)

where R is the salt rejection rate; and C1 and C2 represent the original ion concentration of the salt solution and the ion concentration after filtration, respectively.

## Results and Discussion

### Morphologies of Different GO Composite Membranes

**FIGURE 3:**
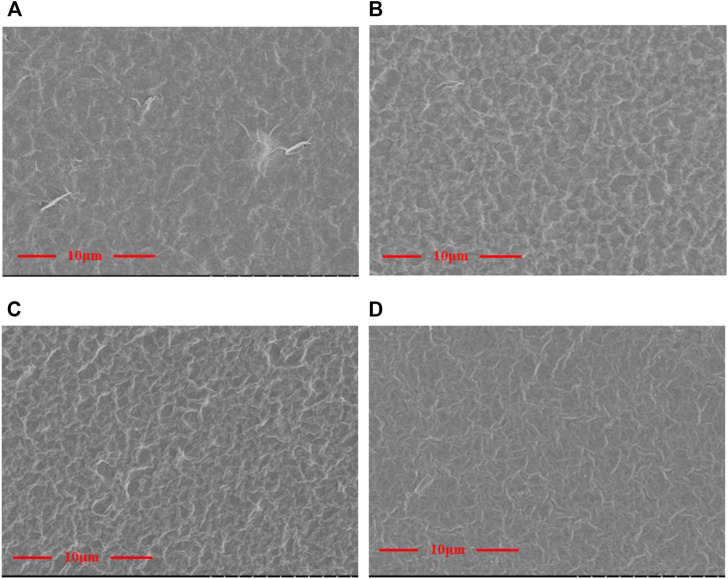
Surface topography of the **(A)** GO/MCE, **(B)** GO-EDA/MCE, **(C)** GO-UR/MCE, and **(D)** GO-PPD/MCE films.

Figure 3 shows the SEM morphology of the GO/MCE, GO-EDA/MCE, and GO-UR/MCE membranes prepared by vacuum filtration using a microporous membrane as the substrate. GO and the composite membranes were slowly deposited on the MCE membrane. The thickness of the GO composite membrane depended on the content of GO in suspension. Figure 3A shows that the GO nanosheets are self-assembled by suspension deposition to form a uniform and dense GO layered membrane structure, which has a flat surface but some small undulations.

The images in Figures 3A–D show that the surface of the cross-linked composite membrane was slightly changed compared to the surface of the GO film. When different kinds of diamine cross-linking agents were introduced, more wrinkles appeared on the surface of the initially flatter GO membrane. The flat GO surface became rough and showed many “valley and ridge” structures.

**FIGURE 4:**
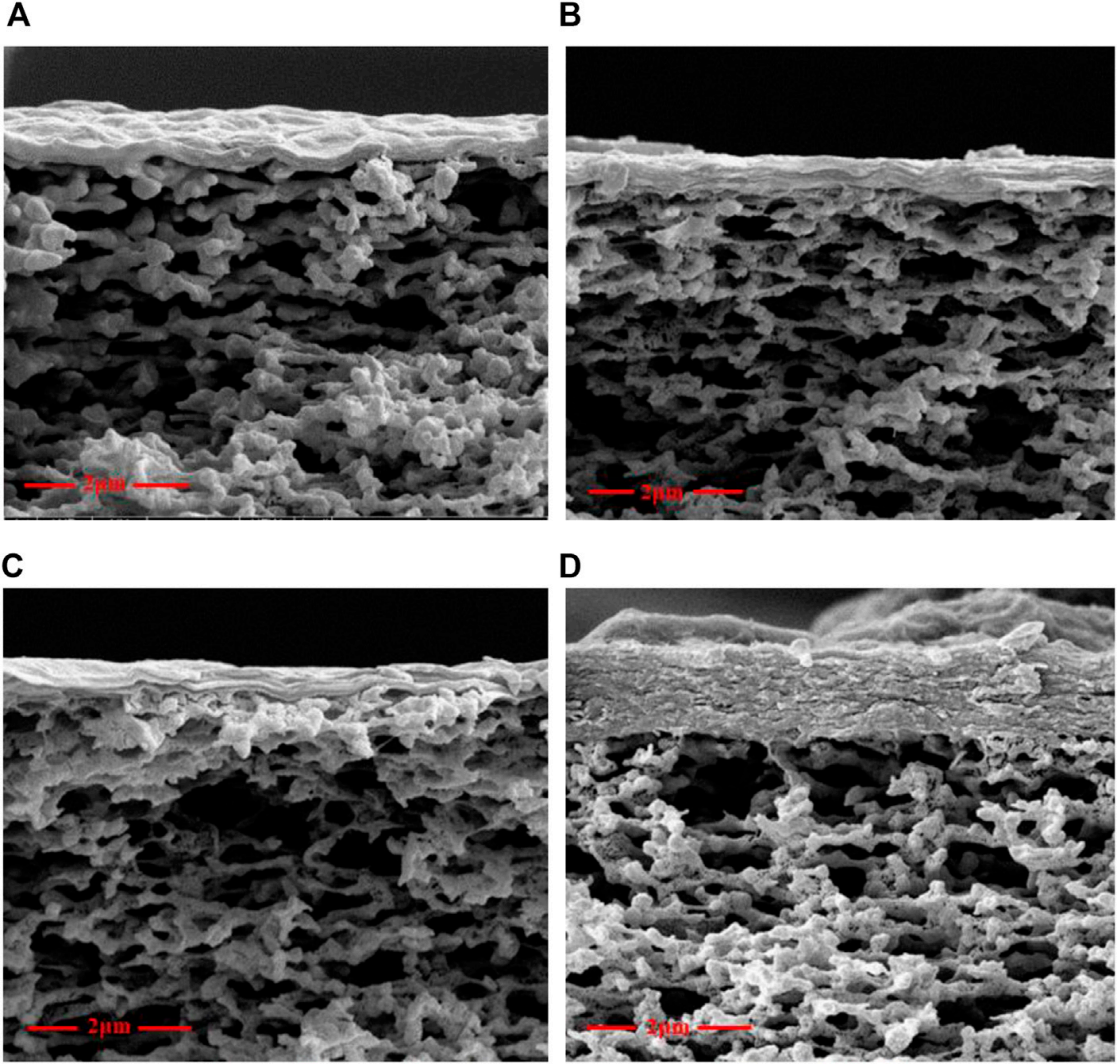
Cross-sectional images of **(A)** GO, **(B)** GO-EDA, **(C)** GO-UR, and **(D)** GO-PPD.

Figure 4 shows a cross-sectional image of the four membranes, wherein the GO layer was self-assembled and attached to the surface of the basement membrane through layer-to-layer pressure. After adding cross-linking agents and a heat treatment, the GO nanoplates stacked closely together to form a dense membrane. The results of XPS and IR tests indicated that the adhesion between the GO layer and MCE support was due to a nucleophilic addition reaction: one end of the diamine monomer reacted with GO (hydroxyl, carbonyl epoxy, and carboxyl), and the other end reacted with MCE (hydroxyl, carbonyl, and acyl); both reactions occurred through covalent bonds. Therefore, in addition to serving as a bridge between the inner layers of GO, the diamine introduces covalent bonds at each end between the modified GO and MCE membranes to maintain stability and a favorable water flux and desalination rate.

In the cross-linking reaction, due to the intervening diamine monomer taking up space between the two layers, it increased the thickness of the composite membrane layer. Furthermore, the increase in the thickness of the GO modified membrane mainly depended on the structure of the diamine monomer. Due to the presence of a benzene ring, the thickness of PPD inserted into a GO layer is larger than that of the other two diamine monomers. The PPD monomer had six carbon aromatic rings and high steric hindrance, which yielded a composite membrane with the thickest layers. In the following section, we will continue to discuss the influence of diamine monomer insertion on membrane thickness, as well as compare the d-spacing and comprehensive performance of the membrane.

### The Structural Characterization of Composite Membranes

**FIGURE 5:**
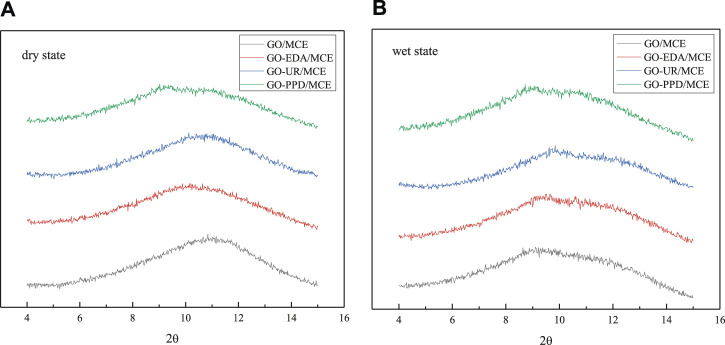
XRD of four composite membranes **(A)** in the dry state, **(B)** in the wet state.

To investigate the structural differences between the GO/MCE, GO-EDA/MCE, GO-UR/MCE and GO-PPD/MCE membranes, different kinds of desalination membranes were tested by XRD. In addition, the Bragg equation was used to calculate the interlayer spacing. Figures 5A shows the XRD results of the four films in a dry state, in which the GO layer (001) peaks are located at 11.040° (GO/MCE, 0.801 nm), 10.185 (GO-EDA/MCE, 0.870 nm), 10.083 (GO-UR/MCE, 0.876 nm), and 9.198 (GO-PPD/MCE, 0.961 nm). The figure and calculation show that after the reaction of GO with EDA or UR, the average GO interlayer spacing was slightly increased compared to that of the GO independent membrane. However, compared with the other two composite membranes, the interlayer spacing of the GO-PPD/MCE membranes increased significantly, which may be caused by the benzene ring structure contained in the PPD monomer. Figures 5B shows the XRD patterns of the four films in the wet state. Due to the introduction of water molecules, the position of the characteristic (001) diffraction peak of the GO composite membrane changed to different degrees, and 2θ decreased to 8.924°, (GO/MCE, 0.991 nm), 9.481 (GO-EDA/MCE, 0.932 nm), 9.341 (GO-UR/MCE, 0.946 nm), and 8.978 (GO-PPD/MCE, 0.984 nm). These results indicated a significant expansion between the GO-independent membrane layers after the introduction of the water molecules. However, the expansion of the membrane after the addition of the cross-linking agent becomes inconspicuous, indicating that the GO layer spacing can be effectively controlled by the cross-linking reaction between the GO nanosheet layers.

In the dry state, not only does GO contain abundant hydrophilic oxygen-containing groups to increase the water flow, but its tighter interlayer channels can provide a great separation efficiency for the GO/MCE membrane. However, GO/MCE has a large layer d-spacing between the sheets in the wet state. The reason for this change is that the GO sheets themselves rely on the delocalized large bonds between the layers themselves for the interlayer connection, and the introduction and penetration of water molecules through the GO layer destroys this weak bond, which leads to the layers being stretched from 11.040 (GO/MCE, 0.801 nm) to 8.92 (GO/MCE, 0.991 nm). Therefore, there is space between the GO layers in the wet state, which can provide a larger nanochannel for molecular or ion permeation. In order to reduce the penetration of ions in the solution filtration process, a cross-linking reaction between different cross-linking agents and the GO layer was selected, which effectively inhibited the expansion of the interlayer d-spacing and further enhanced the ion selectivity of the composite membrane.

**FIGURE 6:**
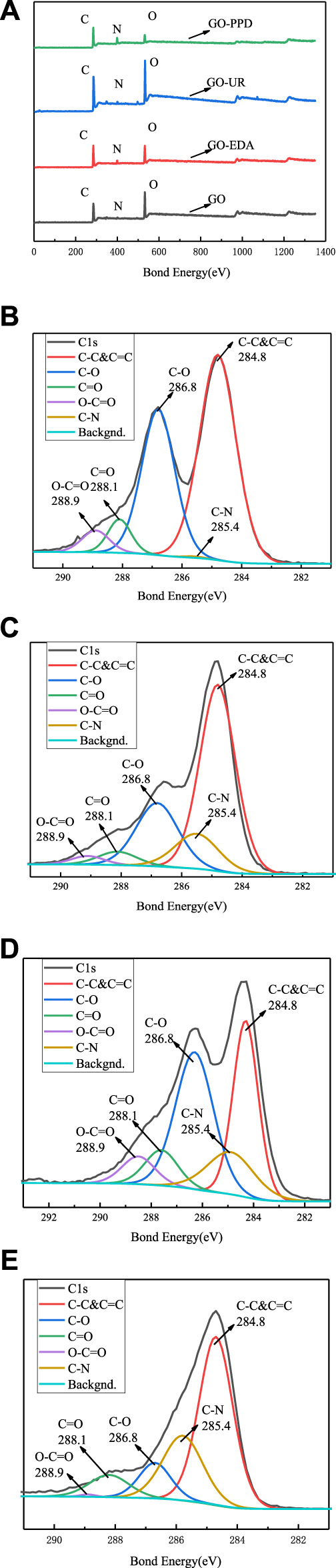
**(A)** XPS total spectrum of the GO/MCE, GO-EDA/MCE, GO-UR/MCE, and GO-PPD/MCE composite membranes; XPS C1s spectrum of the GO/MCE membrane**(B)**, GO-EDA/MCE membrane**(C)**, GO-UR/MCE membrane**(D)**, and GO-PPD/MCE membrane**(E)**.

An XPS elemental analysis chart is shown in Figure 6, and an XPS analysis of the GO/MCE film and the GO-EDA/MCE, GO-UR/MCE, and GO-PPD/MCE composite films is shown in Table 1. After being washing with sufficient deionized water, only chemically bound molecules were present in the composite membrane. The nitrogen content of GO-EDA/MCE, GO-UR/MCE, and GO-PPD/MCE was 6.05, 2.7, and 14.55%, respectively. Due to the reaction consumption between the diamine monomer and the GO oxygen-containing functional group, the proportion of C/O in the GO-EDA/MCE, GO-UR/MCE, and GO-PPD/MCE composite films increased relative to the GO independent film. The GO-PPD/MCE composite membrane had the highest C/O ratio. This was due to the electron withdrawing aromatic ring structure of the PPD, which promoted the reduction reaction between the oxygen-containing functional group of GO and the amino group.

**TABLE 1:** Elemental analysis of GO/MCE, GO-EDA/MCE, GO-UR/MCE, and GO-PPD/MCE films.

Sample	C/%	O/%	N/%	C/O
GO	69.82	30.18	-	2.31
GO-EDA	72.95	21.00	6.05	3.47
GO-UR	69.52	27.78	2.7	2.50
GO-PPD	72.63	12.82	14.55	5.67

Figures 6A shows the XPS spectra of the four membranes, which shows that with the addition of the cross-linking agent, the oxygen-containing group was lost during the reaction, and the C/O ratio in the composite membrane increases. Figures 6C–E show that the C1s epoxy peak intensity decreases with the addition of the cross-linking agent, but a new absorption peak corresponding to a C-N bond appears.

Along with Figures 6C, the XPS results for GO-EDA/MCE (except for the epoxy peak group) shows that the intensity of the diffraction peaks of C=O and COO- decreased significantly, but more C-N bonding occurred. This result indicates that the successful insertion of the diamine monomer consumed a portion of the oxygen-containing group, and the appearance of the C-N absorption peak can also prove that the GO sheets are actually joined together by the cross-linking reaction. According to Figures 6D, the number of oxide groups in the GO-UR/MCE composite membranes is not much less than that for the GO independent membranes, as seen in Figures 6B, and it is higher than that in the GO-EDA/MCE composite membranes. The number of C=O groups in the GO-UR/MCE composite membrane was 1.72 times that of the GO-EDA/MCE composite membrane. The higher content of oxide groups in the GO-UR/MCE composite membrane can be attributed to two factors: the first is that the oxygen-containing functional group was consumed at a lower frequency in the UR cross-linking reaction, and the second is that the addition of UR introduces the C=O group.

The intensity of the diffraction peak of C-O in Figures 6E showed a significant decrease, while the intensity of the C-N peak increased. These changes may be related to the nucleophilic substitution reaction of the three-membered ring that caused the amine to attack GO. As a result, the epoxy ring opened to form a covalent bond between C-N-R and C-OH. Compared with the GO-EDA/MCE and GO-UR/MCE composite membranes, the GO-PPD/MCE membrane showed stronger C-N peaks and smaller C=O peak losses, and its O-C=O peaks disappeared. The reason for this result may be that the amine group reacted with the carboxyl group firstly in PPD, and then with the epoxy group on the edge of GO. The benzene ring and its lone pair of amines in PPD had a high resonance stability. As a result, the reactivity of the amines was low, and the possibility of a reaction with the epoxy groups was small. Furthermore, the amine groups are less reactive in aromatic PPD than in EDA. These diamines tended to donate electrons, resulting in their amines having a high electron density; this formed strong nucleophiles, which preferentially considered the epoxy groups for nucleophilic addition reactions. In addition, it was also possible that the benzene ring of PPD had a large steric hindrance, which neither eroded the carbon on the GO plate nor underwent a ring-opening reaction with the epoxide. Therefore, the reaction between the amines and the carboxyl groups of PPD was the most likely.

The XPS analysis shows that the cross-linking reaction of the diamine monomer consumes a large amount of oxygen-containing functional groups between the GO layers and the edges. At the same time, the corresponding new absorption peak of C-N bond appears and the C/O ratio increases, but the absorption peak intensity of the oxide group decreases sharply. However, under the same conditions, the number of oxide groups in UR is not greatly lost compared to that of the GO and other composite films, and a C-N absorption peak also appears. Therefore, it can be seen that the introduction of UR links the GO nanosheet layers together by a cross-linking reaction, resulting in a decrease in the interlayer spacing, which limits the swelling of the membrane in water. However, the molecular structure can provide a hydrophilic C=O group, so as to achieve better desalination performance. Changes in these factors leads to better desalination performance.

### The Wettability of Composite Membranes

**FIGURE 7:**
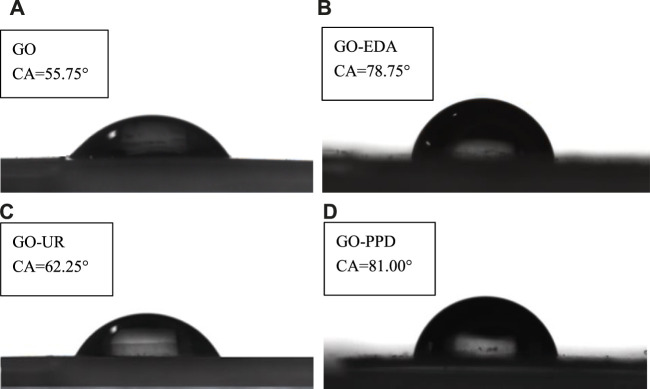
Contact angles of the four membranes with the unfiltered salt solution. **(A)** GO/MCE, **(B)** GO-EDA/MCE, **(C)** GO-UR/MCE, and **(D)** GO-PPD/MCE (dry state).

Figure 7 shows the contact angles (CAs) of the four membranes (dry) with the unfiltered salt solution. The GO film has a contact angle of 55.75 and is hydrophilic. The contact angle of the GO-EDA/MCE membranes is 78.75. The introduction of the cross-linking agent EDA consumed a large amount of oxygen-containing functional groups in GO and increased the contact angle, which lead to the poor hydrophilicity of the GO-EDA/MCE membranes. In addition, the water CA of the GO-PPD/MCE membrane increased to 81.00 due to the hydrophobic aromatic ring structure of PPD and the large consumption of O during the cross-linking reaction. In contrast, the CA of the GO-UR/MCE increased slightly by approximately 6 and the introduction of the cross-linking agent UR has little effect on the hydrophilicity of the GO membrane. The reason for this difference is that the cross-linking agent UR consumes fewer hydrophilic groups. From the XPS analysis, it was known that the UR composite membrane still had a higher O content and more hydrophilic oxygen-containing groups. In addition, the UR monomer has a hydrophilic functional ketone group, which explains why the GO-EDA/MCE membranes have a low permeability volume and why the GO-UR/MCE membranes have a high permeability volume in the desalination test.

**FIGURE 8:**
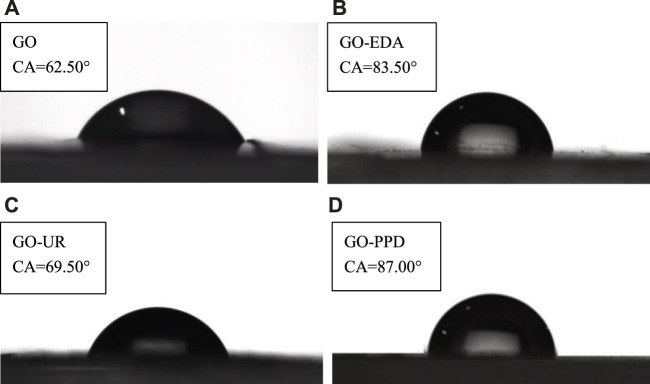
Contact angles of the four membranes that have passed through the filtered salt solution **(A)** GO/MCE, **(B)** GO-EDA/MCE, **(C)** GO-UR/MCE, and **(D)** GO-PPD/MCE (wet state).

Figure 8 shows the contact angles of the four membranes (wet state) that have passed through the filtered salt solution. Compared with the contact angle of the dry membranes, the contact angle of the four membranes increased in the wet state. Since salt ions are continuously deposited on the surface of the membrane during the filtration of the salt solution, the hydrophilic groups on the surface of the film are covered, which caused the hydrophilicity of the film to decrease and the contact angle to increase. However, the influence of the cross-linking agent on the hydrophilicity of the GO membrane did not change, which was consistent with the dry film.

### The Performances of Composite Membranes

This research mainly tested the water flow velocity and ion rejection rate of the GO/MCE membrane, GO-UR/MCE composite membrane, GO-EDA/MCE composite membrane, and GO-PPD/MCE composite membrane in a 0.05 mol/L NaCl solution and CaCl_2_ solution. To obtain accurate experimental data, a positive pressure water penetration test was performed using NaCl, CaCl_2_, and water permeation tests to assess GO/MCE, GO-UR/MCE, GO-EDA/MCE, and GO-PPD/MCE membranes.

**FIGURE 9:**
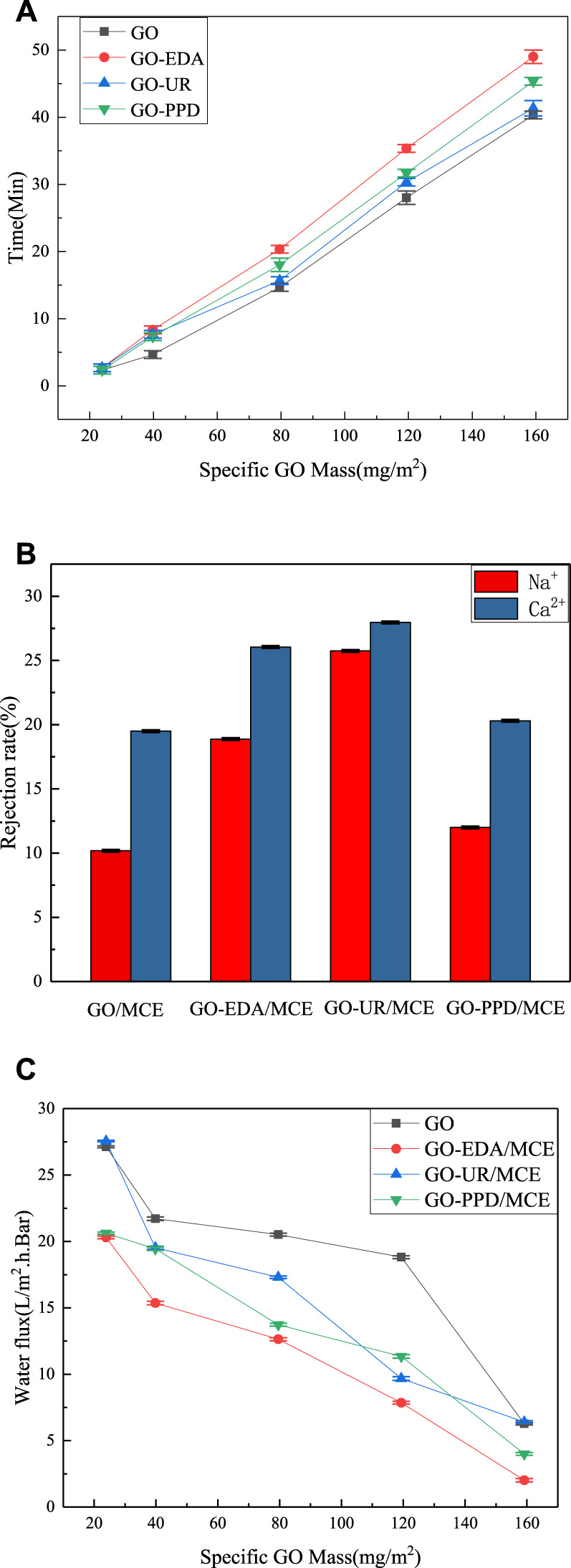
**(A)** Time spent filtering specific GO masses; **(B)** Ion rejection of GO composite membranes; **(C)** Water flux of GO composite membranes with different specific GO qualities.

In the same aqueous solution, different masses of GO were deposited on the MCE membranes with different GO concentrations. Figures 9A shows the time variation of the formation of the composite membrane. When the mass of the GO deposition exceeded 23.87 mg/m^2^, the GO deposition rate dropped substantially. When the mass of the GO deposition is less than 34.5 mg/m^2^, some film defects occur randomly, especially near the film edges. When the GO deposition amount is as high as 39.79 mg/m^2^, a defect-free GO film is obtained on the MCE. Figure 9 shows the results of desalination and water flux of the composite membrane at different deposition mass concentrations. The ion exclusion rate of the GO/MCE membrane is the lowest, and the ion rejection rates of NaCl and CaCl_2_ are 10.18 and 23.80%, respectively. Due to the large interlayer spacing change caused by the benzene ring structure of the PPD monomer, the separation rate of the GO-PPD/MCE membrane was slightly higher than that of the GO/MCE membrane but lower than that of the other two composite membranes. The ion retention of NaCl and CaCl_2_ was 11.99 and 20.30%, respectively. In addition, the separation rate of the GO-EDA/MCE membrane was also higher than that of the GO/MCE membrane, and the ion rejection rates of NaCl and CaCl_2_ were 20.55 and 26.05%, respectively. The layer pitch of the GO-UR/MCE membrane in the wet state was approximately 0.946 nm, while that of GO-EDA/MCE membrane was slightly smaller at 0.932 nm. Nevertheless, the GO-UR/MCE membrane had the better separation performance, and the ion rejection rates of NaCl and CaCl_2_ were 25.74 and 27.96%, which were approximately 1.17–2.53 times higher than those of the GO/MCE membrane, respectively.

**FIGURE 10:**
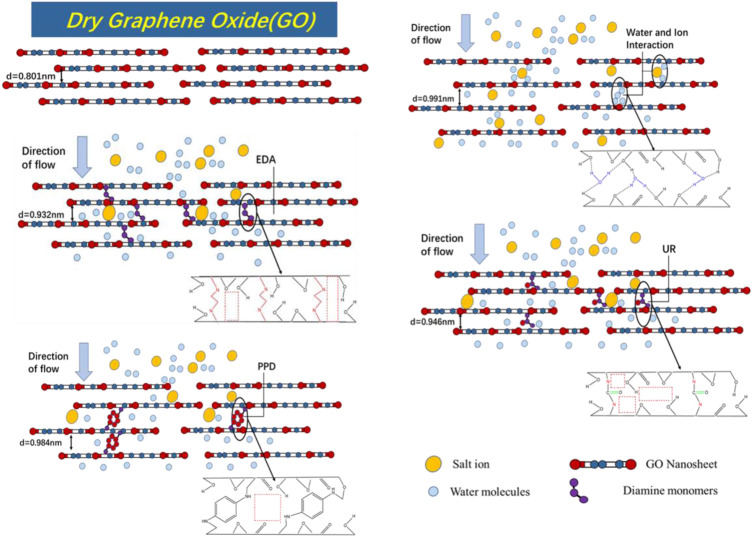
Schematic diagram of GO and composite membrane desalination mechanism and internal physical and chemical structure.

Figures 9C shows the change in water permeability as a function of the specific mass deposition of different amounts of GO. As the amount of GO mass deposition increased, the permeability of all the membranes decreased. The change in the amount of water flow basically corresponded to the change in the CA of the composite membrane. The UR membrane had a water flux similar to the original GO membrane, while the PPD and EDA membranes had a reduced water flux to a certain extent because of their reduced hydrophilicity. However, according to the CA measurement results, it was expected that the hydrophilicity of the PPD membrane decreased the most, yet it showed a higher water flux than the EDA membrane. The reason for this result was that the d-spacing was larger in the wet state, and water molecules passed more easily. These results indicated that the changes in the salt rejection rate and water flux of the GO composite membrane were not only related multiple other factors, but also the result of the interaction between the interlayer distance and the groups in the membrane.

### The Mechanism of Separation and Filtration Over the GO Composite Membrane

The experimental performance test showed that the ion exchange rate of the GO-modified membranes was improved compared with the GO/MCE blank membrane with the introduction of the cross-linking agents EDA, UR, and PPD. The addition of the three diamine monomers showed a certain increase in the interlayer distance, but at the same time, the interlayer distance of the membrane can be kept stable in water.

Due to the presence of benzene rings in the PPD monomer, the GO-PPD/MCE membrane had the largest interlayer distance among the three diamine-monomer-cross-linked composite membranes, which led to the lower ion rejection rate of the GO-PPD/MCE membrane. The hydrophobic aromatic ring structure of the PPD monomer and the large consumption of O during the cross-linking reaction also drastically decreased the hydrophilicity of the membrane. However, XPS and CA tests confirmed that although the PPD membrane had the greatest drop in hydrophilicity, it still showed a slightly better water flux than the EDA membrane. The reason for this was that although the hydrophilic groups between the layers were consumed to a significant extent, the increased interlayer distance still made it easier for water molecules to pass through.

In addition, it was found that the d-spacing of the GO-UR/MCE membrane was larger than the spacing of the GO-EDA/MCE membrane, while the GO-UR/MCE membrane showed a higher ion screening efficiency. These results can be attributed to urea having a ketone group; the ketone group plays an important role in the high repulsion of the GO-UR/MCE membrane. However, the smaller d-spacing of the GO-EDA/MCE film with the lower ion rejection was still beyond our expectations. Compared with UR, EDA had a stronger reducibility. After cross-linking, the amount of oxygen-containing functional groups between GO layers can be consumed by EDA reaction, which significantly reduces the hydrophilicity of the composite membrane. GO-EDA/MCE membrane has lower ion rejection and smaller interlayer d-spacing, which is mainly due to the low number of oxygen-containing groups in GO-EDA/MCE membrane. There was a large amount of free space in the regions without oxygen-containing groups. The reduction of urea was much weaker than that of ethylenediamine, which made it difficult for GO to be reduced by urea. Consequently, the loss of the oxygen-containing groups between GO layers was much lower, which caused the hydrophilicity of the GO-UR/MCE membrane to decrease only slightly. Although the GO-UR/MCE membrane exhibited a large d-spacing in its wet state, it produced a higher ion repulsion, which was necessarily associated with more oxygen-containing groups present in the GO membrane. Comparing the three diamine cross-linkers, it was found that while the d-spacing varied between the GO layers, the functional groups carried by the cross-linker also had a great influence on the structural properties of the membranes. Therefore, controlling the d-spacing and the interaction of the functional groups within the membrane were the two key factors affecting the desalination performance of the GO composite membranes.

## Conclusion

In this experiment, two-layer-based cross-linked composite films based on GO were prepared by adjusting the interlayer spacing of the GO membrane. The variation in the desalting properties of the Na^+^ solution and Ca^2+^ solution between the three membranes at different depositional qualities was investigated.

Compared with the GO membrane, the introduction of the cross-linking agent resulted in more pronounced peak ridges and peaks and valleys on the surface of the composite membrane, indicating that the introduction of the cross-linking agent did indeed cause a shrinkage of the GO sheet layer after self-stacking. The introduction of UR resulted in certain morphological differences that were found through electron microscopy images. The addition of three cross-linking agents did cause some changes to the microstructure of the GO film. We found that the introduction of the cross-linking agent increased the contact angle of the GO film and deteriorated the hydrophilicity. The hydrophilicity of EDA and PPD composite membrane decreased greatly, while the hydrophilicity of UR composite membrane decreased slightly. However, the water flow rate test showed that the GO-UR/MCE composite membrane still maintained a good performance.

After further structural and chemical analysis, it was found that with the introduction of water molecules, there was a significant expansion between the GO independent membrane layers, and the interlayer spacing of the membranes did not change significantly after the addition of the cross-linking agent, which made the membranes have a higher ion selectivity. The introduction of the diamine monomer cross-linker EDA did lose some of the oxygen-containing groups, but the GO sheets were also linked together due to their cross-linking reaction. Although the introduction of PPD monomers further stabilized the interlayer distance, it also consumed a large number of oxygen-containing groups. In addition, because the hydrophobic benzene ring carried by the PPD monomer and the functional group itself occupied a large space, the desalination rate of the membrane was not significantly improved and the water flow rate was decreased. The introduction of the cross-linking agent UR not only reduced the GO sheet spacing, but it also provided a hydrophilic C=O group for the GO film, which resulted in better desalination properties.

In summary, the GO-UR/MCE membranes have a higher ion repulsion and better water flux than the GO membranes. The GO-UR/MCE membranes had a ketone group, which is the main cause of the higher rejection and better water flux. Moreover, there were more oxygen-containing groups in the GO-UR/MCE membrane, which resulted in a small amount free space that prevented the ions from leaching through the GO layer filtration. Although the GO-PPD/MCE membrane exhibited the worst hydrophilicity due to the presence of the hydrophobic benzene ring and the large consumption of oxygen-containing groups between layers, it exhibited the largest interlayer distance in the cross-linked composite membrane, which made its water flux larger than that of the GO-EDA/MCE membrane. Therefore, it was found that one single factor alone cannot effectively control the water permeability and ion rejection rate of the GO composite membrane. Controlling of d-spacing and the combined action of the functional groups in the membrane are both key factors to determine the desalination performance of GO composite membranes.

## Data Availability

The original contributions presented in the study are included in the article/Supplementary Materials, further inquiries can be directed to the corresponding author.
